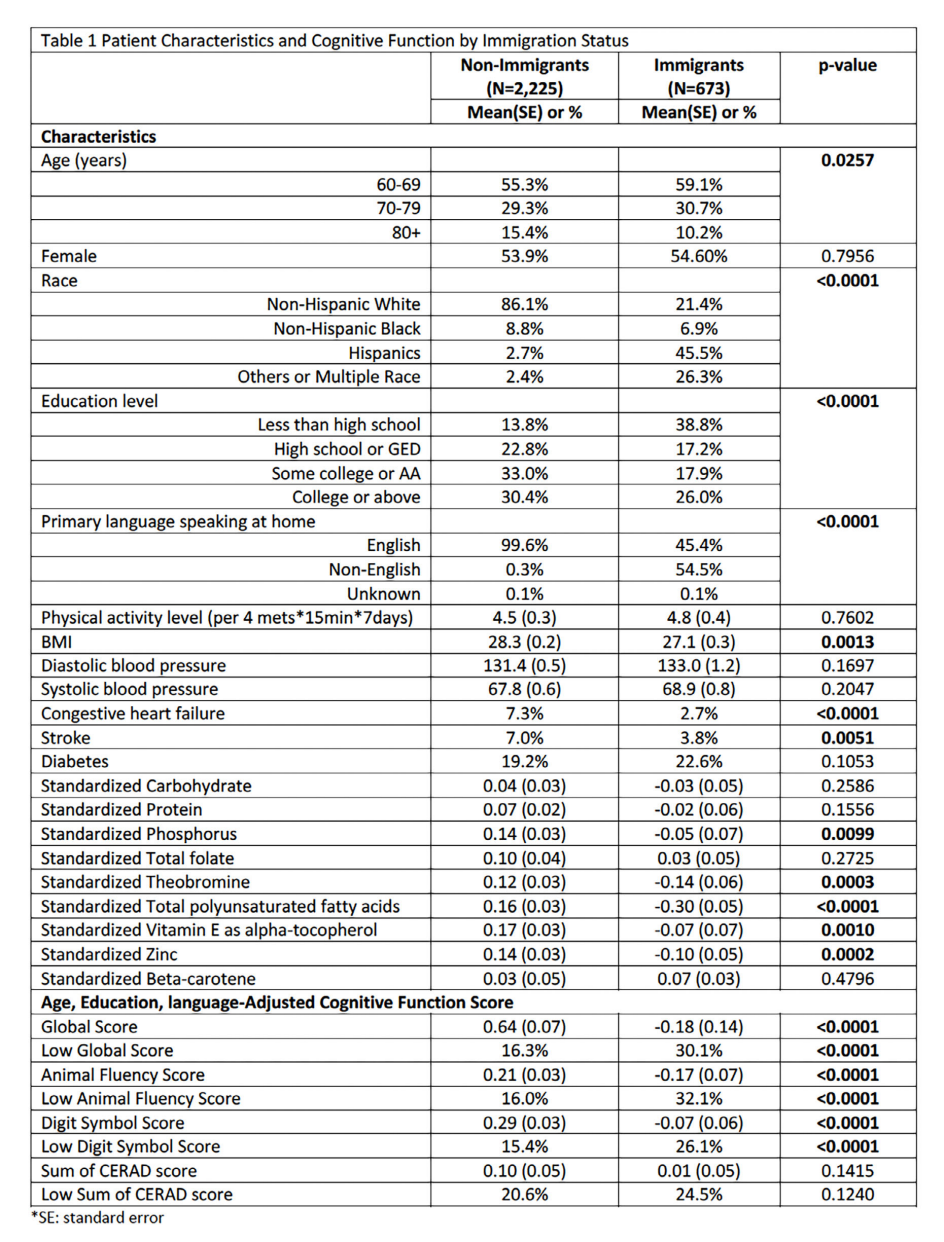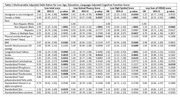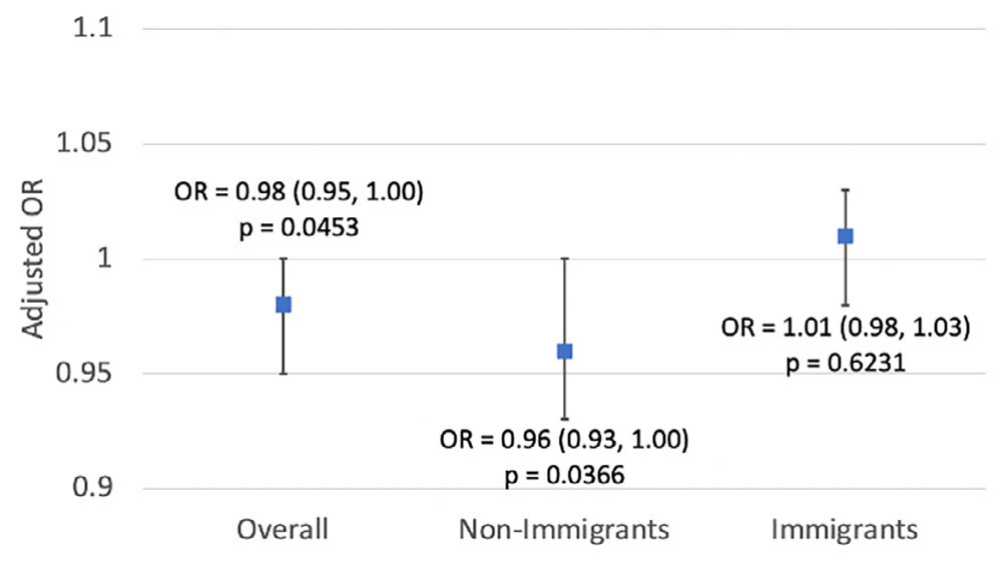# Immigration Status and Cognitive Function among Older Adults

**DOI:** 10.1002/alz.089043

**Published:** 2025-01-09

**Authors:** Yan Cheng, Adnan Lakdawala, Ali Ahmed, Edward Zamrini, Qing Zeng

**Affiliations:** ^1^ George Washington University, Washington, DC USA; ^2^ Washington DC VA Medical Center, Washington, DC USA; ^3^ Irvine Clinical Research, Irvine, CA USA; ^4^ VA Washington DC Healthcare, Washington, DC USA

## Abstract

**Background:**

Prior research on immigration and cognitive functioning was limited with conflicting findings. This study seeks to understand the association between immigration status and cognitive function in a representative sample of the US population and examine physical activity and nutritional intake’s interaction with immigration status.

**Method:**

The study cohort included 2,898 participants (673 immigrants and 2225 non‐immigrants) from US National Health and Nutrition Examination Survey (NHANES) 2011‐2014 who underwent at least one cognitive function test. Demographics, vital signs, cardiovascular diseases, physical activity, and nutrition were compared between immigrants and non‐immigrants by mean or proportion. Standardized z‐scores were calculated for each nutrient to ensure comparability. Cognitive function was assessed through four test scores (animal fluency, digit symbol, Consortium to Establish a Registry for Alzheimer’s Disease [CERAD], and global score). To account for potential cognitive disparities, standardized z‐scores were calculated for cognitive function scores within age, education, and language strata. Scores in the lowest quartile were classified as low. Multivariate logistic regressions were conducted to estimate the association between immigration status and risk of low standardized cognitive function scores, adjusting for all covariates. NHANES survey weights were applied to ensure the results are representative of US civilians.

**Result:**

Compared to the non‐immigrants, the immigrants in our sample were younger and less educated. (Table 1). Our analysis found that being an immigrant was significantly associated with a low global cognitive function score (OR = 2.13, 95% CI = (1.34, 3.40), p = 0.0024) (Table 2). Higher protein, folate and theobromine intakes were associated with a higher global cognitive function score, while higher carbohydrate and zinc intakes were associated with a lower score. No interaction was found between nutrient intakes and immigration status. Increased physical activity was associated with the reduced risk of low animal fluency score, especially beneficial in non‐immigrants (Figure 1).

**Conclusion:**

Being an immigrant was associated with low cognitive function after controlling age, education, language, and other covariates. A higher intake of protein, folate, and theobromine were found to be protective, though they did not have significant interactions with immigrant status.